# Scanning tunnelling microscope light emission: Finite temperature current noise and over cut-off emission

**DOI:** 10.1038/s41598-017-03766-x

**Published:** 2017-06-14

**Authors:** Vijith Kalathingal, Paul Dawson, J. Mitra

**Affiliations:** 1 School of Physics, Indian Institute of Science Education and Research, Thiruvananthapuram, Kerala 695016 India; 2 Centre for Nanostructured Media, Queen’s University, Belfast, BT7 1NN United Kingdom

## Abstract

The spectral distribution of light emitted from a scanning tunnelling microscope junction not only bears its intrinsic plasmonic signature but is also imprinted with the characteristics of optical frequency fluc- tuations of the tunnel current. Experimental spectra from gold-gold tunnel junctions are presented that show a strong bias (*V*_*b*_) dependence, curiously with emission at energies higher than the quantum cut-off (*eV*_*b*_); a component that decays monotonically with increasing bias. The spectral evolution is explained by developing a theoretical model for the power spectral density of tunnel current fluctuations, incorporating finite temperature contribution through consideration of the quantum transport in the system. Notably, the observed decay of the over cut-off emission is found to be critically associated with, and well explained in terms of the variation in junction conductance with *V*_*b*_. The investigation highlights the scope of plasmon-mediated light emission as a unique probe of high frequency fluctuations in electronic systems that are fundamental to the electrical generation and control of plasmons.

## Introduction

**Figure 1:**
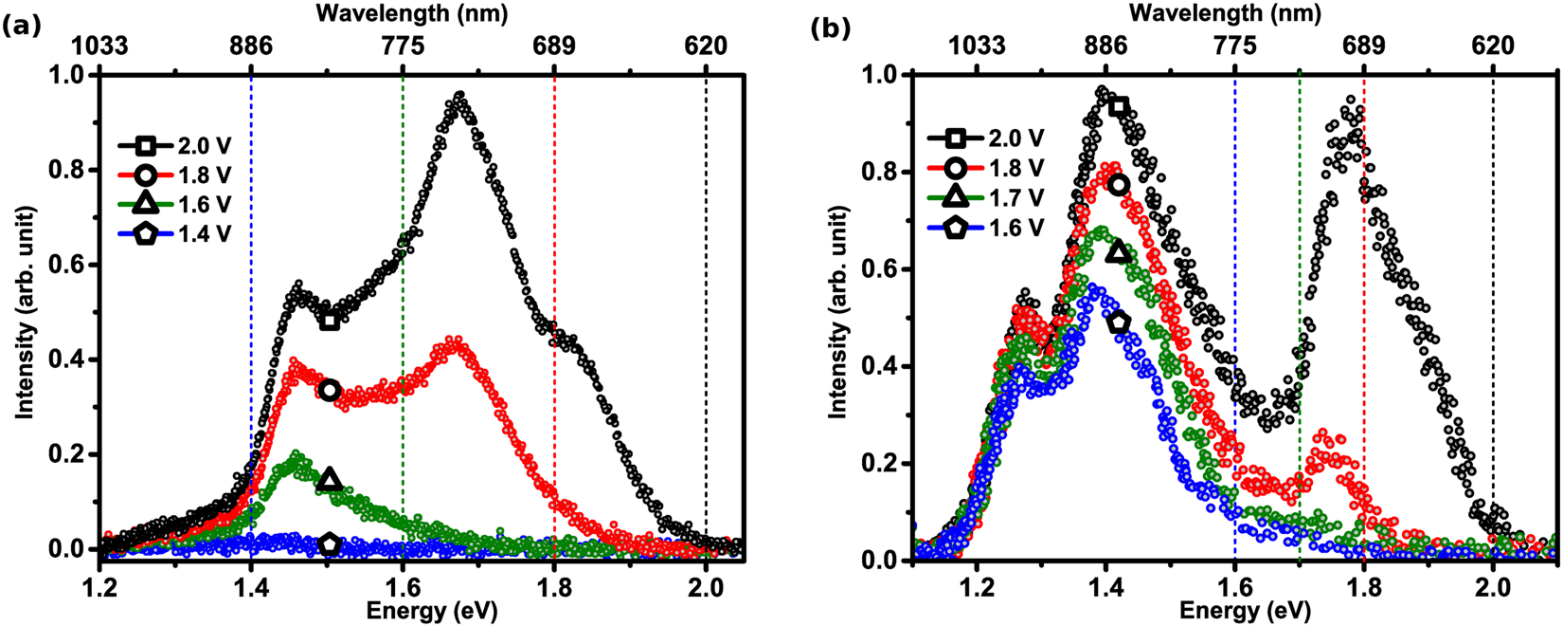
(**a**,**b**) STM light emission spectra recorded with various applied bias, at constant current (~10 nA). Vertical lines demarcate the energies corresponding to *ħω*_*co*_ = *eV*_*b*_.

Plasmonics has long been dominated by the ability of passive structures to concentrate externally incident electromagnetic radiation into volumes of significantly sub-wavelength dimensions^1^. The phenomenon has been exploited in a number of areas, notable examples of which include surface-enhanced Raman spectroscopy^2, 3^, molecular sensing^4^, heat-assisted magnetic recording^5^ and enhanced photovoltaic schemes^6^. However, there has been increasing awareness that the field is lacking without the development of electrically-actuated, active plasmonic devices^7^, where such devices will be critical to the optimal development of on-chip plasmonic circuitry^8–11^. One such class of device is the biased metal-insulator-metal tunnel junction, in which plasmons are generated via inelastically tunnelling electrons. The importance of these devices has been demonstrated through recent experiments into novel tunnel junction architectures^12–14^, displaying their potential in integrating photonics with electronics. From previous experimental^15–23^ and theoretical^24–28^ studies into light emission from tunnel junctions, specifically the scanning tunnelling microscope (STM), it is understood that optical frequency fluctuations in the tunnel current (*I*_*T*_) excite both localized surface plasmons (LSP)^29–31^ as well as propagating surface plasmon polaritons^32–34^. A fraction of these plasmons then radiate into the far-field, emitting photons. Light emission from the STM offers a unique and tunable tool to study the physics and applications of electrical plasmon excitation, where in-spite of the many notable advances^35–38^, significantly more remains unexplained, pertaining to the dynamics and control of the process. A typical emission spectrum, recorded on the junction side of the STM has a broadband background accentuated with distinct peaks at characteristic LSP modal energies, as depicted in Fig. 1, which shows the spectral plots of light emission from Au-Au junctions, recorded experimentally at room temperature. The LSP energies are characteristic of the tip-sample junction (TSJ), determined by its geometry^26, 28, 39^ including the tunnel gap dimension, the dielectric properties of tip and sample^37^ and importantly the local environment^22, 36^. There is a further crucial aspect of the spectral output, related to the intrinsic mechanism of plasmon excitation which is the electrical noise of the driven tunnel junction. The spectral composition of the tunnel current fluctuations carry imprints of the associated energy scales, *k*_*B*_ *T* and *eV*_*b*_, determined by the equilibrium lattice temperature (*T*) and applied bias (*V*_*b*_). The effect of current noise, on the emission spectra, is particularly evident close to the quantum cut-off (*ħω*_*co*_ = *eV*_*b*_), at which excess noise is expected to decrease *linearly* to zero^40, 41^, in the limit *k*_*B*_ *T* ≪ *eV*_*b*_. However, contrary to the intuitive expectation that the maximum energy (*ħω*_*m*_) of emission will also be limited to *ħω*_*co*_, the experimental results show significant emission intensity with energies *ħω* > *ħω*_*co*_, such that *ħω*_*m*_ > *ħω*_*co*_. Importantly, the nature of this over cut-off emission has been distinct in two regimes that are differentiated by the magnitude of either *I*_*T*_, or the junction conductance (*G*_*J*_) that is primarily decided by the tunnel gap (*d*). A consideration of *d* leads us to draw a distinction between a high current (*I*_*T*_ > 10 *μ*A), high *G*_*J*_ regime, comparable to the conductance quantum $({G}_{0}=\frac{2{e}^{2}}{h})$, and a low current (*I*_*T*_ ≤ 100 nA), low *G*_*J*_ (≪*G*_0_) regime. It is only in the former case, where *d*≲ a lattice constant^42–45^, that large violation of the quantum cut-off threshold is observed where photons are emitted with excess energy Δ*E* (=*ħω*_*m*_ − *ħω*_*co*_), with *ℏω*_*m*﻿_ ~ 2*eV*_*b*_. Origins of the above have been attributed to either multi-electron and electron-plasmon interactions^44, 46–49^ or to spontaneous black-body like emission^14, 42^ from a hot electron cloud of local electron temperature between 1000–8000 K. Since light emission originating from multi-electron processes is highly compromised in comparison to those from *1e* processes, due to their significantly lower quantum-efficiency (~10^−7^)^44^, there is a requirement of large *I*_*T*_ for the reliable detection of optical emission significantly above *ħω*_*co*_. Additionally, both multi-electron and electron-plasmon interactions mandate a highly non-equilibrium electron distribution^46^ in the electrodes - departing significantly from the Fermi-Dirac distribution. Plasmonic interaction in this extreme tunnelling to point contact regime is further complicated by quantum plasmonic^50–52^, non-local^53^ and charge transfer effects. By contrast in the low *I*_*T*_ (low *G*_*J*_) regime, emission above *ħω*_*co*_ has been shown to extend typically up to Δ*E* ≈ 150 meV and is characterized by a tail in the emission spectra decaying to zero at a bias dependent maximum. This phenomenon was first explored by Pechou *et al*.^54^ and is evidenced in the spectra shown in Fig. 1, from our own experimental investigations. Here, the spectra are recorded in the visible to near infrared wavelength range (600–1600 nm) from Au-Au TSJs, operating in the ambient with *I*_*T*_ ≤ 50 nA, *V*_*b*_ ≤ 2 V and *G*_*J*_ ~ 10^−5^ *G*_0_. Under these operating conditions the tunnelling electrons redistribute their energy rapidly in the tip and sample material, resulting in electron temperature elevation of ~ few 100’s of K^55–57^. Consequently, we have considered the finite temperature equilibrium Fermi-Dirac electron distribution in the tip and sample in our analysis. We present a model to analyse the *V*_*b*_ dependence of the emission spectra and discuss the evolution of over cut-off emission on the basis of the variation of finite temperature *I*_*T*_ fluctuations with *V*_*b*_. The model has been developed following two classic theoretical treatises, pertaining to electrical transport across metal-insulator-metal tunnel junctions^58–60^ and current noise of a driven tunnel junction^61^, crucially incorporating finite temperature effects and the physics of STM operation. Though developed with reference to light emitted from a STM, the analysis is directly pertinent to break-junctions and other tunnelling devices in general.

## Theory

In order to explain the above-threshold emission reported here it is necessary to consider what is conceptually relevant to the situation - and what is not - before developing a specific theoretical approach. The emission spectra discussed here are considered characteristic of a *1e* excitation process involving excitation of LSPs for the following reasons. First, the emission intensity (ℑ) as reported here (Fig. 1) and in our previous investigations^22, 37^, is dependent on *I*_*T*_ in a slightly super-linear fashion i.e. ${\rm{\Im }}\propto {({I}_{T})}^{\beta },(\beta \simeq 1)$. This is consistent, for example, with the *1e* emission reported by Schull *et al*.^44^ for which *β* = 1.1 and contrasts with the *2e* emission where *β* = 1.7. Moreover, to state the obvious, any *2e* mechanism will give rise to photons of energy significantly in excess of the linear *eV*_*b*_ threshold, extending up to energies ~2*eV*_*b*_. Here, the experimentally detected maximum violation of the quantum cut-off threshold is *ħω*_*m*_ ≈ 1.1*eV*_*b*_. The second salient point is that the spectral structure in Fig. 1 is characteristic of an emission mechanism driven by the excitation and decay of junction LSPs. That the multi-peak spectral profile is not consistent with black-body like spectra and that there is only a modest temperature increase, under the STM operating conditions, leads us to conclude that a component of black-body radiation can not solely account for the spectral tail extending above *eV*_*b*_. The exclusion from consideration of *e*-*e* interactions, black body radiation and the quantum plasmonic regime, associated with massive wavefunction overlap at gap dimensions of the order of the lattice constant, constrains us to analysis in terms of a perturbative linear theory.

The overall shape of the emission spectra (Fig. 1) is a convolution of two quantities, (a) the intrinsic plasmonics of the junction, and (b) the energetics of the plasmon excitation trigger i.e. *I*_*T*_ fluctuations, quantified by their power spectral density (*P*_*I*_ (*ω*)). While the former’s contribution is evidenced as peaks at characteristic LSP energies dictated by the geometry and optical properties of the TSJ material, the latter is intrinsic to the tunnelling process, per se. Current fluctuations associated with the quantum transport across tunnel junctions are understood to reflect the inelastic tunnelling processes, which are strongly coupled to the electromagnetic environment. It is interesting to note that investigations into the inverse effect i.e. photon induced electron emission, provide additional information regarding hot-electron distribution and electron-photon coupling in such systems^62, 63^. Noise in electronic systems has been extensively investigated only in the low to medium frequency range (≤100 GHz). A regime where the overall noise, in general, is dominated by the absorptive component of the electromagnetic coupling^64^. By contrast emission from tunnel junctions, particularly from STM TSJs provides a unique platform to evidence and investigate noise associated selectively with the emission component of the electromagnetic coupling and importantly at visible frequencies (~100 s of THz), which is impractical with purely electronic devices. The experimentally observed quasi-linear dependence of ℑ (*ω*) on *I*_*T*_ reflects the analogous dependence of *P*_*I*_ (*ω*) on *I*_*T*_, as stated by the fluctuation-dissipation theorem^65^ that relates fluctuations in a system to its linear response, extended to the case of a driven tunnel junction^40, 41, 61, 64^. Experimentally, *I*_*T*_ noise is evidenced as shot, thermal and 1/*f* noise, at various frequency (energy) regimes. While the 1/*f* noise^66^ is exclusive at *f* < 10 Hz, shot noise^67^ scales with the total current and is most evident when thermal^68^ noise is suppressed e.g. at low temperatures. Theoretically, the quantum cut-off limit is imposed on the emission through *I*_*T*_ dependent *P*_*I*_ (*ω*), which is analysed here for a biased TSJ at finite temperatures. To derive an analytic expression for the non-linear *I*_*T*_ (*V*_*b*_), we follow Simmons’ classic analysis of electrons tunnelling between two metallic electrodes separated by a insulating gap of width *d* ^59, 60^.

For tunnelling between two electrodes (1 and 2), the number of electrons (*N*_+_) tunnelling from electrode 1 to electrode 2, with the former held at ground potential and the latter at a ‘positive’ *V*_*b*_ and the reverse (*N*_−_) are given as^59^;

1 $${N}_{\pm }=\frac{4\pi {m}^{2}}{{h}^{3}}{\int }_{0}^{{E}_{m}}D({E}_{x}){f}_{\pm }({E}_{x})d{E}_{x}$$

where *m* is the electron mass, *h* is the Planck’s constant, ${E}_{x}=m{v}_{x}^{2}\mathrm{/2}$ is the kinetic energy of an electron along the tunnel direction (*x*), perpendicular to the electrodes and *E*_*m*_ is the maximum energy of the tunnel electrons (~barrier height). *D*(*E*_*x*_), the tunnelling probability is obtained using WKB approximation as^60^;

2 $$D({E}_{x})\approx \exp \,(-A\sqrt{\overline{\varphi }-\frac{e{V}_{b}}{2}})\,\exp \,(-\frac{A({\varepsilon }_{f}-{E}_{x})}{2\sqrt{\overline{\varphi }-\frac{e{V}_{b}}{2}}})$$

where $\overline{\varphi }$ is the mean barrier height and $A=4\pi d\sqrt{2m}/h$ and *ε*_*f*_ is the equilibrium Fermi level. Equation (2) is valid under the condition $({\varepsilon }_{f}-{E}_{x})\ll \overline{\varphi }$. For a finite *T* calculation it is important to consider the ${f}_{\pm }({E}_{x})$ terms that originate from partially integrated Fermi-Dirac functions, integrated over energies (*E*_*r*_) associated with components perpendicular to the tunnel direction and are given as;

3 $${f}_{+}({E}_{x})={\int }_{0}^{\infty }f(E)d{E}_{r}={k}_{B}T\,\mathrm{ln}(1+\exp \,(\frac{{\varepsilon }_{f}-{E}_{x}}{{k}_{B}T}))$$

4 $${f}_{-}({E}_{x})={\int }_{0}^{\infty }f(E+e{V}_{b})d{E}_{r}={k}_{B}T\,\mathrm{ln}(1+\exp \,(\frac{{\varepsilon }_{f}-{E}_{x}-e{V}_{b}}{{k}_{B}T}))$$

where, ${E}_{r}=m({v}_{y}^{2}+{v}_{z}^{2}\mathrm{)/2}$. Consequently, the forward and reverse current densities may be written as;

5 $${J}_{\pm }=\frac{4\pi me}{{h}^{3}}\,\exp \,(-A\sqrt{\overline{\varphi }-\frac{e{V}_{b}}{2}})\times {\int }_{0}^{\infty }{f}_{\pm }({E}_{x})\,\exp \,(-\frac{A({\varepsilon }_{f}-{E}_{x})}{2\sqrt{\overline{\varphi }-\frac{e{V}_{b}}{2}}})d{E}_{x}$$

where the integral upper limit, *E*_*m*_, in Eq. (1) has been extended to infinity since ${E}_{m}\gg {k}_{B}T$ for *T* ≲ 300 K, which gives the net current density *J* = *J*_+_ − *J*_−_ as^60^;

6 $$J({V}_{b},T)=\frac{4\pi me}{{h}^{3}{B}^{2}}\frac{\pi B{k}_{B}T}{\sin (\pi B{k}_{B}T)}\,\exp (-A\sqrt{\overline{\varphi }-\frac{e{V}_{b}}{2}})\times \mathrm{(1}-\exp \,(-Be{V}_{b}))$$

where, $$B=\frac{A}{2\sqrt{\overline{\varphi }-\frac{e{V}_{b}}{2}}}.$$

The inherent current fluctuations of a tunnel junction or the current noise is calculated in the steady state as^61^;

7 $${P}_{I}\,(\omega )={|I(\omega )|}^{2}={{\rm{\Sigma }}}_{f}{|\langle f|\hat{I}|i\rangle |}^{2}\delta (\omega -({E}_{f}-{E}_{i})/\hslash )$$

The current operator $\hat{I}$ in Eq. (7) transfers electrons from an initial state (|*i*〉) of electrode 1 to a final state (|*f*〉) in electrode 2 and back. The spectral weight of fluctuations corresponding to the photon emission part of *P*_*I*_ (*ω*), and thus emission from tunnel junctions carrying a current *I* at finite *T*, is given by Eq. (8). The symmetric term related to photon absorption from the electromagnetic environment is neglected henceforth.

8 $${P}_{I}\,(\omega )=\frac{e}{2\pi }[I(e{V}_{b}-\hslash \omega )\,\coth (\frac{e{V}_{b}-\hslash \omega }{2{k}_{B}T})]$$

For a STM TSJ at *T* = 0 K and $e{V}_{b}\ll \overline{\varphi }$, such that the current, $I\simeq {V}_{b}/{R}_{eff}$, where *R*_*eff*_ is the effective junction resistance, Eq. (8) simplifies to;

9 $${P}_{I}\,(\omega {)|}_{e{V}_{b}\ll \overline{\varphi }}=\frac{e{V}_{b}}{2\pi {R}_{eff}}(1-\frac{\hslash \omega }{e{V}_{b}})$$

yielding the zero temperature quantum cut-off condition^24^, ensuring that all emission due to *1e* processes are quenched at *ħω* = *eV*_*b*_. It quantifies the presence of current fluctuations originating exclusively from the driven nature of the junction, devoid of any thermal contribution. An expression for *P*_*I*_ (*ω*) of a biased TSJ at finite *T* is then obtained from Eq. (8) in conjunction with that for *J* (Equation (6)) as given in Eq. (10).

10 $${P}_{I}\,(\omega )=\alpha \frac{2\pi m{e}^{2}}{{h}^{3}{B}^{^{\prime} 2}}\frac{B^{\prime} {k}_{B}T}{\sin (\pi B^{\prime} {k}_{B}T)}{e}^{-A\sqrt{\overline{\varphi }-\frac{(e{V}_{b}-\hslash \omega )}{2}}}\times \{1-{e}^{-B^{\prime} (e{V}_{b}-\hslash \omega )}\}\coth (\frac{e{V}_{b}-\hslash \omega }{2{k}_{B}T})$$

where, $B^{\prime} =\frac{A}{2\sqrt{\overline{\varphi }-\frac{e{V}_{b}-\hslash \omega }{2}}}$ and *α* denotes the effective area of the TSJ and is taken to be unity in our calculations. It is worth reiterating that *P*_*I*_ (*ω*) (Equation (10)), quantifies the strength of the *I*_*T*_ fluctuations at energy *ħω*, which stimulates the junction LSPs and ultimately decides the emission intensity. It is dependent on the tunnel gap, *V*_*b*_, electronic properties of TSJ material and *T*. The finite *T* effects get incorporated into the above expression through the functions ${f}_{\pm }({E}_{x})$ (Equations (3) and (4)) along with the *T* dependence built into Eq. (10).

## Results and Discussion

The experimentally recorded emission spectra from two different Au-Au STM junctions are shown in Fig. 1(a,b), for various values of *V*_*b*_ ranging from 1.4 to 2.0 V, in steps of 0.2 V. Experimental details have been published elsewhere^22, 28, 39^. The plots are uncorrected for the spectral response of the Si CCD, which dictates the lower energy cut-off (~1.1 eV) of the spectra. For recording each spectrum *I*_*T*_ was held constant (~10 nA) at a particular *V*_*b*_. In Fig. 1(a), the spectrum for *V*_*b*_ = 1.4 V is devoid of any specific features though the output is distinctly non-zero around 1.4 eV. Increasing *V*_*b*_ then progressively reveals peaks corresponding to the LSP modes of the TSJ. The spectrum for *V*_*b*_ = 2.0 V shows three spectral peaks around 1.8, 1.7 and 1.5 eV that were not evidenced at lower biases. Further, the emission intensity is seen to increase with increasing *V*_*b*_ at all energies. A similar spectral evolution, with *V*_*b*_ is observed for the second Au-Au TSJ, shown in Fig. 1(b). The coloured dashed lines in the figures, matching the colour of a corresponding spectrum, demarcate the *ħω*_*co*_ = *eV*_*b*_ point for the respective spectrum. The demarcations clearly show that emission is not quenched at the respective *ħω*_*co*_ but goes to zero (i.e. reaches the detector noise floor) at *ħω*_*m*_, extending to energies significantly beyond *eV*_*b*_. Interestingly, while the emission intensity in this regime increases with *V*_*b*_, the over cut-off energy span (Δ*E*) actually decreases with increasing *V*_*b*_. From Fig. 1(a) Δ*E* is obtained as ~0.20, 0.15 and 0.05 eV for *V*_*b*_ = 1.6, 1.8 and 2.0 V, respectively with comparable values extracted from Fig. 1(b) and previous reports^14, 54^. Further, the overall maximum energy of light emission from a Au-Au TSJ is likely to be limited by the onset of inter-band transitions ~2.4 eV [Experimentally, the overall light emission detection window is decided by the photo-detector threshold and onset of the inter-band transitions in case of Au with *V*_*b*_ further limiting the emission to ~*ħω*_*co*_ + Δ*E*].

**Figure 2:**
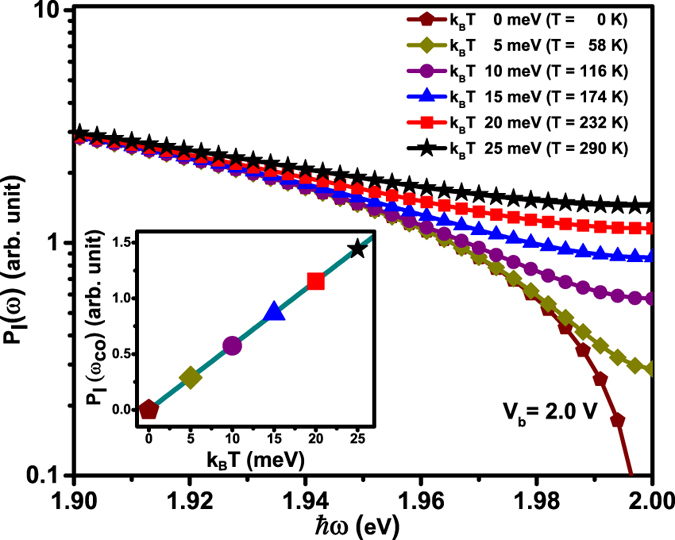
Semi-log plot of energy dependence of *P*_*I*_ (*ω*) for biased tunnel junction for various *k*_*B*_ *T* (inset: temperature variation of *P*_*I*_ (*ω*_*co*_) at *ħω*_*co*_ = 2 eV).

In order to understand the effect of various parameters that affect the emission intensity via *P*_*I*_ (*ω*) we firstly analyse the effect of finite *T*. Figure 2 shows the semi-log plot of the numerically calculated *P*_*I*_ (*ω*) using Eq. (10), for a Au-Au junction ($\overline{\varphi }=5.1$ eV) at a fixed *V*_*b*_ = 2.0 V and *d* = 0.63 nm with *k*_*B*_ *T* varied from 0–25 meV (in steps of 5 meV), i.e. from absolute zero to 290 K. As seen in the context of our previous investigations^22, 28, 37, 39^ the tunnel gap of a STM operating with *I*_*T*_ ≤ 50 nA and *V*_*b*_ ≤ 2 V, typically ranges between 0.6–1.0 nm. [All numerical calculations have been performed using *Wolfram Ma﻿thematica* ^﻿®^﻿10].﻿ Evolution of the curves with increasing *T* shows that the finite *T* significantly affects *P*_*I*_ (*ω*) in the regime *ħω* → *eV*_*b*_. At *ħω*_*co*_, *P*_*I*_ (*ω*) saturates to a finite background noise floor, unlike the zero *T* case where it goes to zero, identically. Figure 2 also shows that difference between the curves, calculated at different *T*, becomes negligible for lower energy fluctuations away from *eV*_*b*_. Inset of Fig. 2 plots the *T* dependence of *P*_*I*_ (*ω*_*co*_) i.e. for *ħω* = *eV*_*b*_, which is analytically given by Eq. (11).

11 $${P}_{I}\,({\omega }_{co})\propto \frac{{({k}_{B}T)}^{2}}{\sin (\frac{A\pi }{2\sqrt{\overline{\varphi }}}{k}_{B}T)}$$

For the TSJ parameters given above, the argument of the sine function = 4.273*k*_*B*_ *T* indicates that *P*_*I*_ (*ω*_*co*_) ∝ *k*_*B*_ *T*, which is reminiscent of Johnson noise. Importantly, the *T* dependent plots show that as *ħω* → *eV*_*b*_ the contribution of driven current noise, arising due to finite *V*_*b*_, to the total current noise is substantially reduced and *P*_*I*_ (*ω*), in this regime, becomes dominated by current noise originating from finite *T* effects, progressively becoming stronger with increasing *T* ^67^. This non-zero residual current noise is the likely source of the finite emission at energies *ħω* ≥ *eV*_*b*_, observed experimentally.

**Figure 3:**
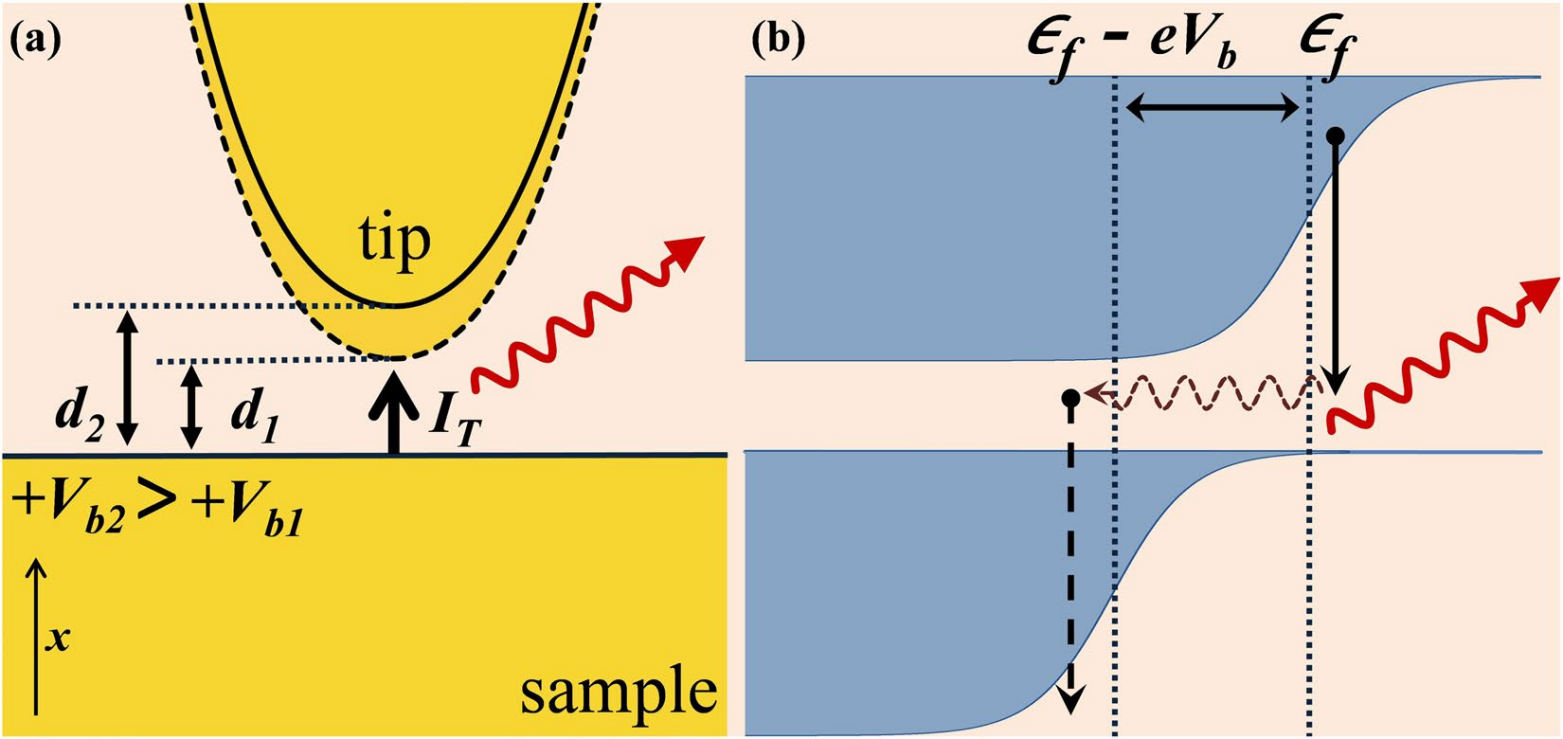
(**a**) Schematic of STM junction showing variation of tunnel gap with changing bias, keeping current constant. (**b**) Tip and sample Fermi functions at finite *T*, showing inelastic tunnelling of thermally excited electron from tip to sample giving rise to emission *ħω* > *eV*_*b*_.

**Figure 4:**
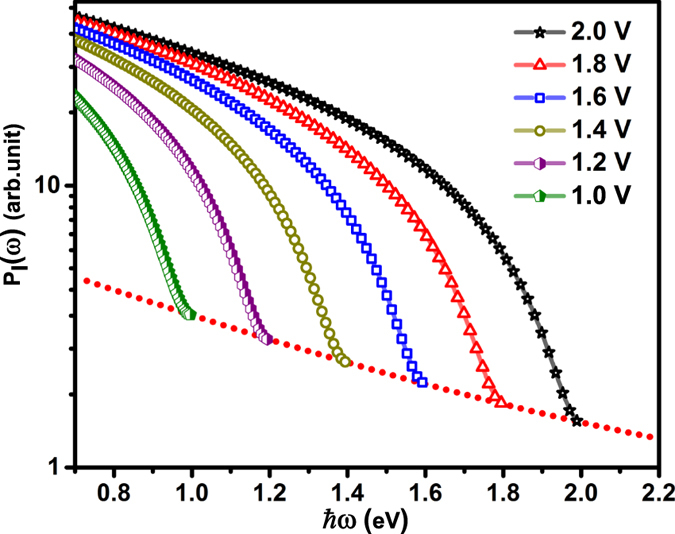
Semi-log plot of *P*_*I*_ (*ω*) *vs*. energy (for *k*_*B*_ *T* = 25 meV) for various applied bias between 1–2 V (Red dotted line shows an exponential fit to residual *P*_*I*_ (*ω*) at *ħω*_*co*_ = *eV*_*b*_).

**Figure 5:**
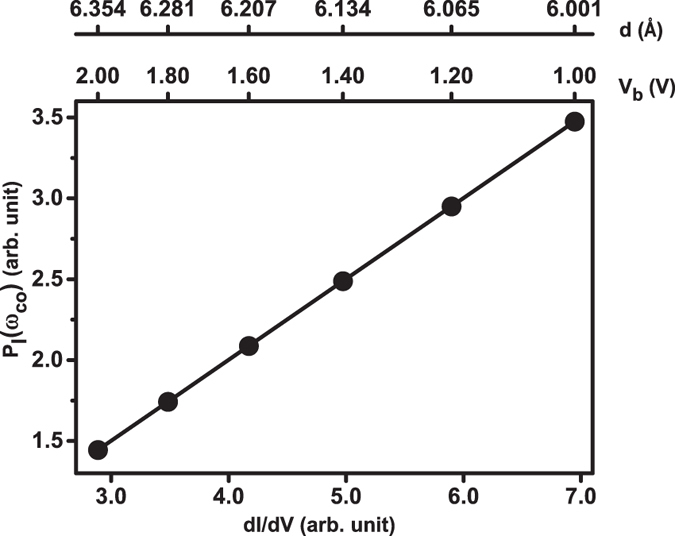
Variation of residual *P*_*I*_ (*ω*_*co*_) with junction conductance. Top *x* axes gives the corresponding values of *V*_*b*_ and tunnel gap *d*.

The current-voltage relation, Eq. (6), of a given STM TSJ is mediated by tunnel gap (*d*). Operationally, if *V*_*b*_ across the TSJ is increased (decreased) keeping *I*_*T*_ constant, the feedback increases (decreases) *d* to compensate the change by effectively decreasing (increasing) *G*_*J*_ of the TSJ. The effect is shown schematically in Fig. 3(a). Thus, merely decreasing *V*_*b*_ at constant *I*_*T*_ also increases *G*_*J*_, unlike that in a break junction where the *I* − *V* characteristics are linked by a unique transfer function. Figure 4 plots the variation of *P*_*I*_ (*ω*) with *ħω* for various values of *V*_*b*_ maintaining *I*_*T*_ constant, for *k*_*B*_ *T* = 25 meV. In calculating the above spectra the current component in Eq. (10) is held constant by adjusting *d* to compensate for the changing *V*_*b*_. Here, change in *V*_*b*_ from 2.0–1.4 V is accompanied by a decrease in *d* in the range 0.63–0.6 nm. The spectra show that, for each *V*_*b*_, as *ħω* → *eV*_*b*_, *P*_*I*_ (*ω*) saturates to a minimum *V*_*b*_ (and T) dependent noise floor *P*_*I*_ (*ω*_*co*_). The important point to note here is that each *P*_*I*_ (*ω*) plot, corresponding to a specific *V*_*b*_, is distinctly different even though the magnitude of *I*_*T*_ is constant. The difference between the spectra again is most prominent close to their respective quantum cut-off and diminishes at lower energies. Equation (12) gives the finite *T* variation of *P*_*I*_ (*ω*_*co*_) with *d*, i.e. at *ħω* = *eV*_*b*_.

12 $${P}_{I}\,({\omega }_{co})=\alpha \frac{4\pi m{e}^{2}\,{({k}_{B}T)}^{2}}{{h}^{3}}\frac{\exp (-A\sqrt{\bar{\varphi }})}{\sin (A\frac{\pi {k}_{B}T}{2\sqrt{\overline{\varphi }}})}$$

where the parameter *A* is linearly dependent on *d* indicating a dominantly exponential dependence of *P*_*I*_ (*ω*_*co*_) on *d*. The inherent inter-dependence between *V*_*b*_ and *d* thus results in a systematic variation of *P*_*I*_ (*ω*_*co*_) with *V*_*b*_ as shown by the red dotted line in Fig. 4. The above dependence is also reflected in the scaling of *P*_*I*_ (*ω*_*co*_) with *G*_*J*_ (Fig. 5), calculated from Eq. (6) in which the top axis gives the corresponding *V*_*b*_ and *d* values used in the calculation.

**Figure 6:**
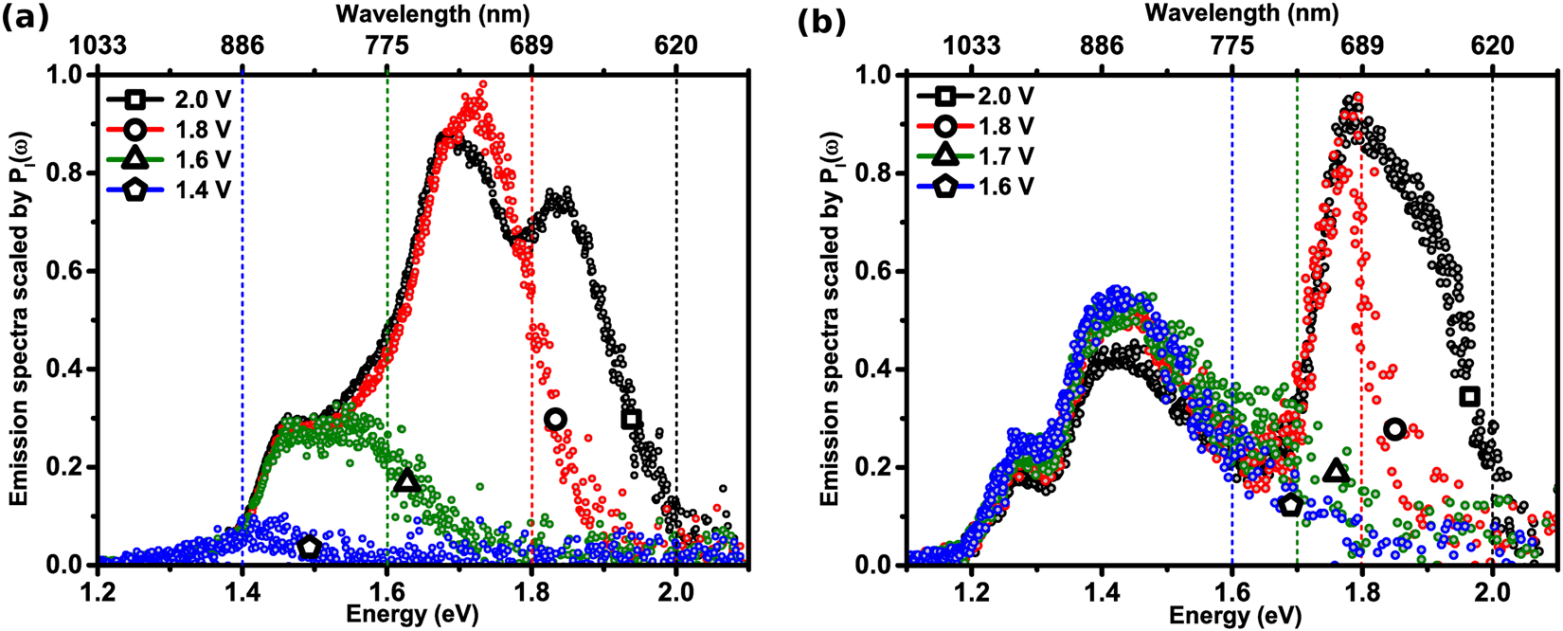
(**a**,**b**) Light emission spectra at various biases, scaled with the corresponding tunnel current power spectral density.

To re-iterate, the overall shape of the emission spectra (Fig. 1) is analytically a product of the energetics of the junction LSP modes and *P*_*I*_ (*ω*) dependent on the parameters discussed above^22^. Operationally, as the junction *V*_*b*_ is increased it progressively expands the energy window of “allowed” light emission in an experiment, systematically evidencing the LSP modes lying energetically within the allowed widow as distinct peaks in the emission spectrum. Thus, if the individual spectra acquired at the various *V*_*b*_ are scaled with the corresponding calculated *P*_*I*_ (*ω*), they should collapse onto a single curve, mimicking the intrinsic LSP contribution, neglecting the small variation in LSP modal energies due to change in *d*. Figure 6 shows the scaled spectra for the two Au-Au TSJ data presented in Fig. 1. Data in the range *ħω* > *ħω*_*co*_ has been scaled by *P*_*I*_ (*ω*_*co*_). Evidently, the various spectra scale onto a ‘single’ curve in support of the above theory. Its worth noting that the low temperature scaling curve (Equation(9)) has been used previously to model the STM emission spectra^22^. At this stage of investigation we ascribe the differences between the scaled spectra to experimental detection or temporal drift error in acquiring the spectra sequentially. See Section II in Supplementary Information for an alternative analysis of the above scaling and additional details.

**Figure 7:**
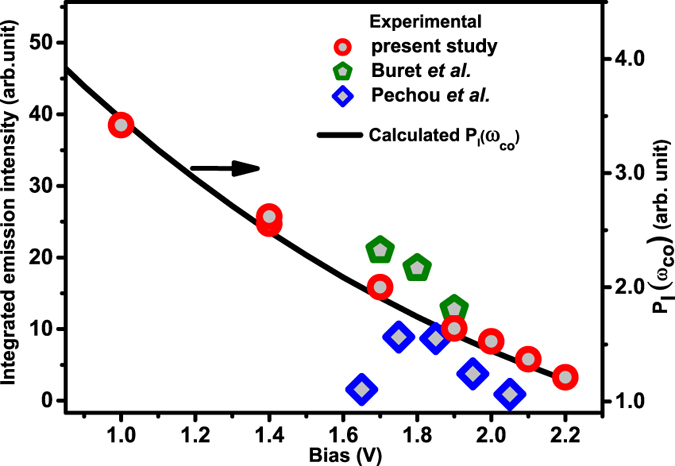
Variation of the integrated light emission intensity above the quantum cut-off with applied bias (data from refs 14, 54 is also included). Solid line shows the variation of residual *P*_*I*_ (*ω*_*co*_) with bias.

Equations (9) and (12) indicate that while the strength of *I*_*T*_ fluctuations at 0 K for a biased tunnel junction goes to zero at *ħω*_*co*_, at finite *T* it saturates to *V*_*b*_ dependent residual noise floor, which may stimulate junction plasmons and thus far-field light emission, at and above *ħω*_*co*_. Within the scope of the present analysis involving *1e* tunnelling, emission with energies higher than *ħω*_*co*_ readily arises from inelastic transfer of tip electrons tunnelling from the filled Fermi tail, say with initial energy *E*_*i*_ > *ε*_*f*_ to an empty state, of the sample, with energy *E*_*f*_ < (*ε*_*f*_ − *eV*_*b*_). Figure 3(b), schematically depicts one such process, arising from thermal smearing of the Fermi distribution about *ε*_*f*_ that results in energy loss (*E*_*i*_ − *E*_*f*_) larger than *ħω*_*co*_. Evidently for any given emission energy (*ħω* = *E*_*i*_ − *E*_*f*_) there exists multiple combinations of initial and final states that may give rise to emission with *ħω* > *ħω*_*co*_. However, the number of such available combinations decrease steeply as the *ħω* becomes larger than *ħω*_*co*_, thus giving rise to the decaying intensity tail of over cut-off emission, evidenced in each individual spectrum in Fig. 1. Pertinently, the residual noise floor, *P*_*I*_ (*ω*_*co*_), shows a specific functional dependence on *V*_*b*_ (Figs 4 and 5), which must be mimicked by that of the over cut-off emission, if the former is the source of the latter. Figure 7 plots the *V*_*b*_ dependence of integrated emission intensity for *ħω*_*co*_ ≤ *ħω* ≤ *ħω*_*m*_ emission from Au-Au junctions obtained at constant *I*_*T*_ = 10 nA and *V*_*b*_ varied between 1.0–2.2 V. The plot also includes integrated emission intensity data, in the same spectral range, from STM junctions reported by Pechou *et al*.^54^, with *I*_*T*_ = 10 nA and from break-junctions reported by Buret *et al*.^14^, for low conductance junctions of *G*_*J*_ ~ 0.01*G*_0_. The black line shows the residual noise power, *P*_*I*_ (*ω*_*co*_), calculated for various *V*_*b*_. The highly comparable variation of both quantities with *V*_*b*_ gives credence to the conjecture that they are intimately linked to each other, specifically that *P*_*I*_ (*ω*_*co*_) is a measure of the strength of over cut-off emission. The earlier reported generic decrease of the over cut-off emission energy span Δ*E* with increasing *V*_*b*_ is also consistent with the above decrease in *P*_*I*_ (*ω*_*co*_) strength with *V*_*b*_.

**Figure 8:**
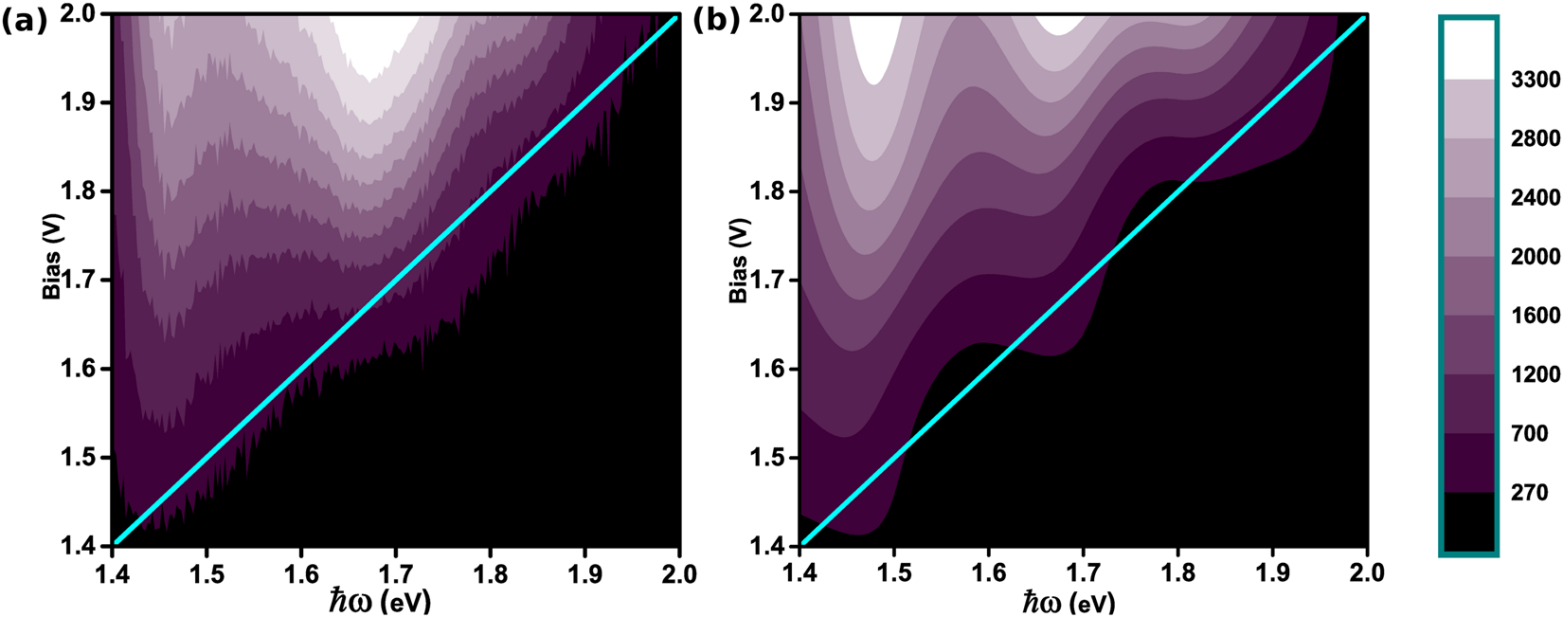
Contour plots of bias and energy dependent emission intensity from a Au-Au STM junction with constant tunnel current (~10 nA) (**a**) experimentally obtained from spectra in Fig. 1(a), and (**b**) theoretically calculated using *P*_*I*_ (*ω*) (Equation (10)) (see Section I, Supplementary Information for further details). Scale bar indicates the emission intensity in false colour. Cyan line corresponds to *eV*_*b*_ = *ħω*_*co*_ which represents the limit of *1e* processes.

To further elucidate the efficacy of the model developed to describe the experimental observations, especially in the over cut-off regime, we have theoretically calculated the STM emission spectra in terms of the *P*_*I*_ (*ω*) (Equation (10)) and the plasmonics of the junction (i.e. the LSP modes) following the phenomenological model of Rendell and Scalapino (RS)^24^ and later expanded by Boyle *et al*.^22^ (see Section I, Supplementary Information for details of the LSP mode calculation). Figure 8(a) shows the emission intensity contour plot generated from the experimental STM emission spectra shown in Fig. 1(a), as a function of *V*_*b*_ and *ħω*. Figure 8(b) shows the corresponding theoretically calculated emission intensity contour plot. While the overall similarity between the LSP modal energies recorded experimentally and reflected in the theoretical plots are obvious from Figs 8 and S1, S2 (Supplementary Information), it is worth noting the effect of finite temperature contributions in the theoretical calculations. The cyan lines in Fig. 8 denote the quantum cut-off condition *V*_*b*_ = *ħω*_*co*_/*e* and the finite emission intensity contours extending to the right of the cyan lines correspond to over cut-off emission, observed experimentally and calculated theoretically, which would otherwise be absent for a *T* = 0 K calculation of *P*_*I*_ (*ω*). It is important to note that while inclusion of the finite *T* effect, in calculating *P*_*I*_ (*ω*) ensures a non-zero, finite noise power at the quantum cut-off, the strength of *P*_*I*_ (*ω*_*co*_) is crucially dependent on the junction conductance and therefore applied bias.

## Conclusions

In conclusion, we have obtained a straightforward analytic expression to calculate current noise of low conductance STM TSJs, crucially including finite *T* effects. The calculated noise power, *P*_*I*_ (*ω*) showed characteristic dependencies on the relevant energy scales decided by *T* and applied bias, allowing us to investigate the role of *P*_*I*_ (*ω*) as the stimulator of junction plasmons and the ensuing light emission, especially with energies in excess of the quantum cut-off. The investigation shows that even in the regime *k*_*B*_ *T* ≪ *ħω*, where bias driven current noise is expected to be the dominant source of excitation of junction plasmons, finite *T* effects play a significant role in determining *P*_*I*_ (*ω*). The finite *T* contributions are especially significant at energies close to the quantum cut-off, where the finite residual current noise *P*_*I*_ (*ω*_*co*_) provides a direct source of over cut-off emission originating solely from *1e* processes, neglecting the lower probability multi-electron processes or black-body like emission from hot electrons. Though the theoretical framework presented here is developed on the basis of light emission spectra recorded from a STM operated in the ambient, the analysis is equally applicable to emission from other tunnel devices, allowing better comprehension of the multi-dimensional parameter space in optimizing their plasmonic response, especially the role of junction bias in active plasmonics. Finally, the study also showcases the relevance of the light emission phenomenon to investigate noise in electronic systems at the ultra high frequency (hundreds of terahertz) regime.

### Data Availability

The datasets generated and / or analysed in the current study are available from the corresponding author on reasonable request.

## Electronic supplementary material

Supplementary Information: Scanning tunnelling microscope light emission: Finite temperature current noise and over cut-off emission
