# Supplementary material for: Scanning tunnelling microscope light emission: Finite temperature current noise and over cut-off emission
Source: Sci Rep. 2017 Jun 14;7:3530. doi: 10.1038/s41598-017-03766-x (PMC5471255; doi:10.1038/s41598-017-03766-x)
Supplement: Supplementary file 1 — Supplementary Information: Scanning tunnelling microscope light emission: Finite temperature current noise and over cut-off emission [file 41598_2017_3766_MOESM1_ESM.pdf]

Supplementary Information: Scanning tunnelling microscope light emission: Finite temperature current noise and over cut-off emission

Vijith Kalathingal,[1] Paul Dawson,[2] and J. Mitra[1]

[1]School of Physics, Indian Institute of Science Education and Research, Thiruvananthapuram, Kerala, 695016, India

[2]Centre for Nanostructured Media, Queen's University, Belfast BT7 1NN, United Kingdom

I. COMPARING THE THEORETICAL AND EXPERIMENTAL STM LE SPECTRA

The spectral distribution of the emitted light is a convolution of the tunnel current power spectral density, $P_I(\omega)$ and the intrinsic plasmonics of the tip-sample junction delineated by junction LSP modes[1]. While the finite temperature $P_I(\omega)$ is given by Eq. 10, the latter i.e. the LSP modes may be calculated from the phenomenological model of Rendell and Scalapino (RS)[2] and applied to STM LE by Boyle *et al.* The plasmonic response of the TSJ is modulated by the local geometry and dielectric properties of the TSJ, which has been shown experimentally and modelled theoretically,[1,3–5]. In the RS model, the plasmonic TSJ is approximated as a spherical metal particle of radius R (representing the tip end radius) located at a height d above a flat sample surface, thus constituting a nanosphere-plane (NS-P) system. The plasmonic resonant frequencies (ω_n) of the NS-P system i.e. the LSP modes are then given by,

$$\omega_n = \omega_p \left[\frac{\tanh(n+\frac{1}{2})\beta_0}{\epsilon + \tanh(n+\frac{1}{2})\beta_0} \right]^{1/2} \qquad n = 0, 1, 2.. \tag{S1}$$

where $\beta_0 = \cosh^{-1}(1 + d/R)$, ϵ is the dielectric constant of the medium surrounding the TSJ and n denotes the mode number. In deriving the expression the tip and sample metal dielectric function is described by the Drude free electron model[4–6] The above analytic expression is applicable in the limit $d/R \ll 1$, which is satisfied for most of the STM TSJs[4], where $d \sim 0.5$ nm and $R > 10$ nm. Boyle *et al.*[1] later expanded the RS model to theoretically calculate the entire emission spectrum by modelling the emission as that from a Hertzian dipole located at the TSJ (oscillating with frequency ω). Then for a given TSJ and STM operating parameters, the over all emission intensity $\Im(\omega)$ may be written as[1]:

$$\Im(\omega) = C \times P_I(\omega) \times \sum_{n=0}^{\infty} \frac{\omega^4}{({\omega_n}^2 - \omega^2)^2 - \omega^2\gamma^2} \tag{S2}$$

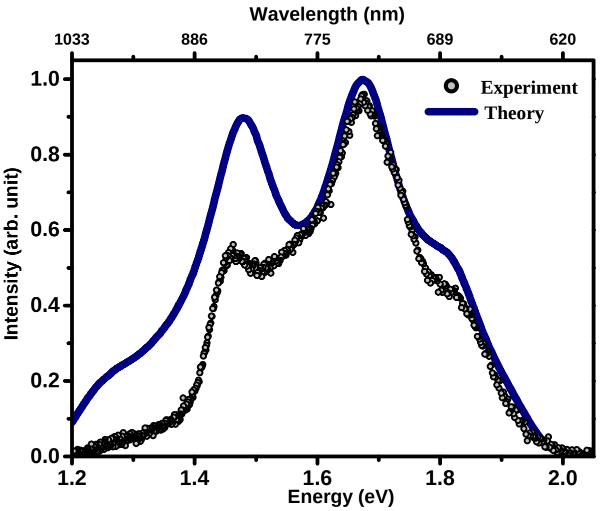

Figure S 1. The experimentally obtained STM LE spectrum from a Au-Au junction for I_T= 10 nA and V_b= 2.0 V and the corresponding theoretical fit using Eq. S2.

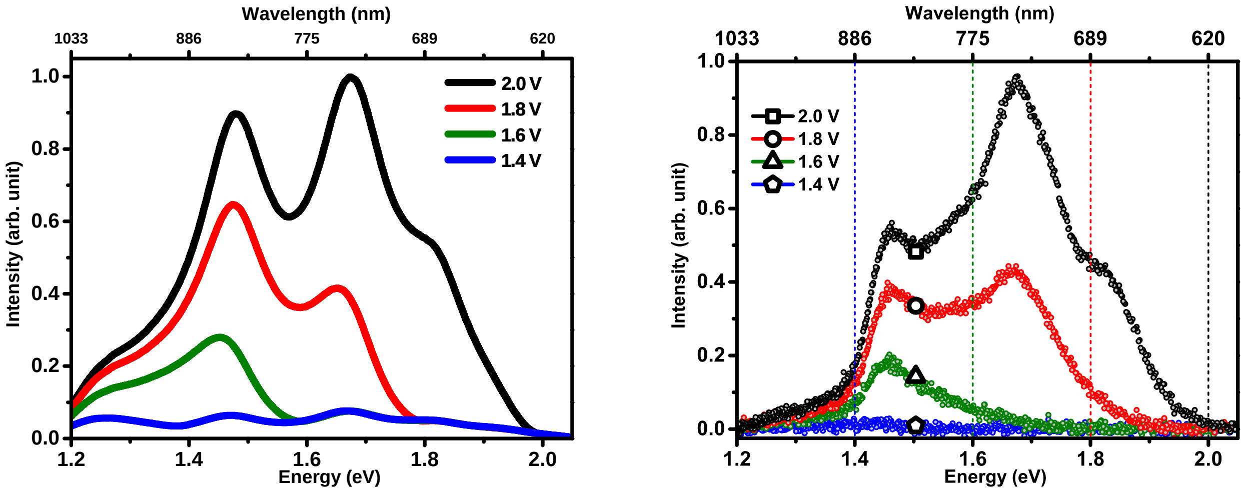

(a) (b)

Figure S 2. (a) Theoretically calculated light emission spectra for a Au-Au STM junction for various junction bias at constant tunnel current. (b) Experimentally obtained STM LE with various applied bias, at constant current ($\sim$ 10 nA).

where C is an arbitrary scaling constant and γ is the damping coefficient for the tip and sample material i.e. Au.

We have used Eq. S2, with $P_I(\omega)$ calculated for V_b = 2.0 V to fit the experimental emission spectrum presented in Fig. 1a for 2.0 V bias as shown in Fig. S1. The best fit geometric parameters are obtained as R = 160 nm for d = 0.63 nm (from Fig 5), yielding the LSP modal energies as $\hbar\omega_2$= 1.48 eV, $\hbar\omega_3$= 1.68 eV, and $\hbar\omega_4$= 1.83 eV, which correspond to the spectral peaks evidenced in Fig. S1. Using the above parameters that generate the calculated spectrum in Fig. S1, the emission spectra corresponding to the other biases are generated, *only* by changing the $P_I(\omega)$ corresponding to V_b, along with the change in d commensurate with V_b necessary for keeping I_T constant (Fig. 5). Figure S2a shows the family of $\Im(\omega)$ plotted as a function of energy ($\hbar\omega$), for various V_b. LSP resonance energies (ω_n) are distinctly visible in the calculated spectra corresponding to the experimental observations re-plotted in Fig. S2b from STM LE spectra plotted in Fig. 1a. For emission at energies $\leq eV_b$ (V_b varied from 2.0 – 1.4 V), Eq. 10 is used to evaluate the $P_I(\omega)$. Above eV_b, exponentially decaying form of the $P_I(\omega)$ given by Eq. 12 is used to include the over cut-off contributions. Overall, for a given STM tunnel junction (with all fit parameters held constant) the V_b dependent variation in the $P_I(\omega)$, accurately reproduces the experimentally obtained STM LE spectrum, as evidenced in Fig S2. These sets of data have been then used to generate the contour plots presented in Fig. 8.

II. VALIDITY OF THE MODEL

In Fig. 6 the various spectra obtained for V_b in the range 1.4 – 2.0 V collapse onto a single curve when scaled with $P_I(\omega)$ for the corresponding V_b. The validity of the above analysis and understanding may be effected in an alternate analysis also. The bare plasmonic response of the TSJ may be obtained by calculating the ratio of emission spectra and the calculated $P_I(\omega)$ at any bias. We have scaled LE spectrum at 2.0 V with the corresponding $P_I(\omega)$ to generate this bare plasmonic response and then used it to predict the expected emission spectra for other biases by multiplying it with $P_I(\omega)$ at those biases as given below.

$$\Im(\omega, V_b) = \frac{Experimental\ Spectra\ at\ 2\ V}{P_I(\omega, 2V)} \times P_I(\omega, V_b) \tag{S3}$$

Emission spectra thus generated for 1.8 V and 1.6 V, by this method are plotted in Fig. S3a and Fig. S3b respectively (black dotted curves) along with the corresponding actual experimental spectra from Fig. 1 (red dotted curves). Again the calculated spectra qualitatively follows the experimental results and confirms the self-consistency of the model and underlying physics discussed here.

The relevance of obtaining an expression for $P_I(\omega)$ including finite T correction in contrast to the zero T calculation (Eq. 9) is elucidated in Fig. S4. It plots the experimental emission spectrum recorded with V_b=1.8 V at 300 K along with the expected spectra at lower temperatures k_BT = 0.1 meV (T=1.2 K) and 10 meV (T=116 K). The expected

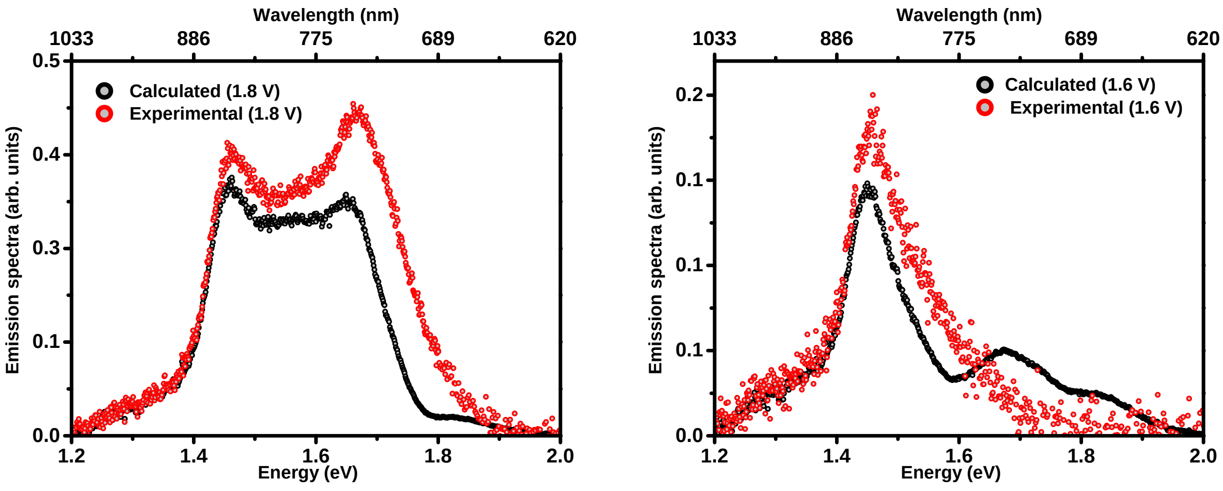

(a) (b)

Figure S 3. Theoretically scaled emission spectra (following Eq. S3) for (a) V_b = 1.8 V and (b) 1.6 V. The experimental spectra are co-plotted for comparison.

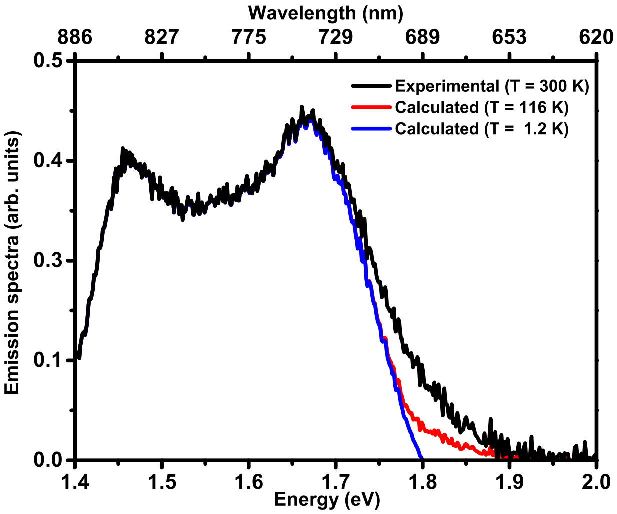

Figure S 4. Experimental spectra for V_b= 1.8 V at 300 K and theoretically scaled emission spectra (following Eq. S4) for T= 116 K and 1.2 K.

spectra at lower temperatures are calculated as;

$$\Im(\omega, T, 1.8\ V) = \frac{Experimental\ Spectra\ at\ 300\ K(1.8\ V)}{P_I(\omega, 300\ K, 1.8\ V)} \times P_I(\omega, T, 1.8\ V) \qquad \text{(S4)}$$

The spectra at 1.8 V was chosen to demonstrate the temperature dependence since it has a well developed emission span both below and above the quantum cut-off. It is evident from the plot that for very low temperatures (k_BT = 0.1 meV), the emission should quench at eV_b, following the conventional zero temperature power spectral density (Eq. 9). With increase in temperature residual thermal noise in the system increases, contributing to the emission beyond the cut-off, consistent with experimental observation.

---

[1] Boyle, M. G., Mitra, J. & Dawson, P. The tip-sample water bridge and light emission from scanning tunnelling microscopy. *Nanotechnology* **20**, 335202 (2009).

[2] Rendell, R. W. & Scalapino, D. J. Surface plasmons confined by microstructures on tunnel junctions. *Phys. Rev. B* **24**, 3276–3294 (1981).

[3] Aizpurua, J., Apell, S. P. & Berndt, R. Role of tip shape in light emission from the scanning tunneling microscope. *Phys. Rev. B* **62**, 2065–2073 (2000).

[4] Mitra, J., Feng, L., Boyle, M. G. & Dawson, P. Electromagnetic interaction between a metallic nanoparticle and surface in tunnelling proximity-modelling and experiment. *J. Phys. D: Appl. Phys.* **42**, 215101–215101 (2009).

[5] Kalathingal, V., Dawson, P. & Mitra, J. Scanning tunneling microscope light emission: Effect of the strong dc field on junction plasmons. *Phys. Rev. B* **94**, 035443 (2016).

[6] Ashcroft, N. W. & Mermin, D. N. *Solid state physics* (Thomson Press Ltd., India, 2003).
